# Prevalence and risk factors of tuberculosis among people living with HIV/AIDS in China: a systematic review and meta-analysis

**DOI:** 10.1186/s12879-023-08575-4

**Published:** 2023-09-06

**Authors:** Cong-Cong Qi, Li-Ran Xu, Chang-Jia Zhao, Hai-Yan Zhang, Qing-Ya Li, Mei-Jun Liu, Ye-Xuan Zhang, Zhou Tang, Xiu-Xia Ma

**Affiliations:** 1 Henan University of Chinese Medicine, Zhengzhou, Henan Province China; 2 Key Laboratory in Chinese Medicine for the Prevention and Treatment of Viral Diseases in Henan Province, Zhengzhou, Henan Province China; 3 The First Affiliated Hospital of Henan University of Chinese Medicine, Renmin Road 19, Jinshui District, Zhengzhou City, Henan Province 450000 China; 4 The First Affiliated Hospital of Henan University of Chinese Medicine, Zhengzhou, Henan Province China

**Keywords:** HIV/AIDS, Tuberculosis, Prevalence, Risk factors, Meta-analysis

## Abstract

**Objective:**

To estimate the prevalence and risk factors associated with tuberculosis (TB) among people living with human immunodeficiency virus (HIV) infection/acquired immunodeficiency syndrome (AIDS) in China.

**Methods:**

A systematic review and meta-analysis were conducted according to the Preferred Reporting Items for Systematic Reviews and Meta-analyses guidelines. After the literature was screened based on the inclusion and exclusion criteria, STATA^®^ version 17.0 software was used for the meta-analysis. The heterogeneity among study data was assessed using *I*^*2*^ statistics. Subgroup analysis and meta-regressions were performed to further explore the source of heterogeneity.

**Results:**

A total of 5241 studies were retrieved. Of these, 44 studies were found to be eligible. The pooled prevalence of HIV/TB co-infection was 6.0%. The risk factors for HIV/TB co-infection included a low CD4^+^ T cell count, smoking, intravenous drug use and several other sociodemographic and clinical factors. Bacillus Calmette–Guérin (BCG) vaccination history was a protective factor.

**Conclusion:**

A high prevalence of TB was observed among people living with HIV/AIDS in China. Low CD4^+^ T cell count, smoking, and intravenous drug use were the primary risk factors for HIV/TB co-infection, whereas BCG vaccination history was a protective factor. Checking for TB should be prioritized in HIV screening and healthcare access.

**Systematic review registration:**

Registered on PROSPERO, Identifier: CRD42022297754.

**Supplementary Information:**

The online version contains supplementary material available at 10.1186/s12879-023-08575-4.

## Introduction

Human immunodeficiency virus (HIV) infection is a major global public health issue, with 650 000 deaths from HIV-related causes reported in 2021 [1]. According to the latest data released by the World Health Organization (WHO), tuberculosis (TB) accounts for approximately 30% of the 690 000 acquired immunodeficiency syndrome (AIDS)-related deaths worldwide [2]. TB remains the leading cause of death among people living with HIV (PLHIV). PLHIV are 15–21 times more likely to develop active TB than people without HIV [2]. A close relationship exists between AIDS and TB. For example, HIV infection can cause TB progression from the latent phase to the active phase and increase the incidence of TB [3]. TB can also promote disease progression in PLHIV and affect the treatment effect, quality of life, and survival duration of patients with HIV/AIDS [4, 5]. HIV and TB are not simply a combination of diseases; rather, they interact to accelerate disease progression. HIV/TB co-infection has become an urgent public health problem.

China has a large number of PLHIV and is one of the high-burden countries for TB. Epidemiological research has shown the presence of 1.14 million PLHIV [6], 58 5340 new cases of TB, and 10 000 cases of HIV/TB co-infection in China [7]. HIV infection greatly increases the risk of developing TB even before CD4 + T-cell counts decrease [8]. Therefore, it is particularly important to control dual HIV/TB infection. Although the mechanism underlying HIV/TB mutual promotion has been elucidated partly in recent years and major breakthroughs have been made in the treatment [9], the low positive rates of acid-fast staining in sputum smears and tuberculin test for HIV/TB co-infection and the prevalence of drug-resistant TB remain major public health challenges. Identifying the risk factors and protective factors for HIV combined with TB, improving TB screening methods, and facilitating early anti-TB are the key to preventing dual infection and reducing mortality.

HIV infection results in immune homeostasis perturbations, which is characterized by CD4 + T-cell depletion and immune activation [10]. High incident TB among HIV-positive patients was estimated, especially in patients with CD4 + T cell count < 200 cells/mm + ^3^ [11]. The case notification rate was higher among male and eldery PLHIV [12, 13], Individuals aged 35–44 years constituted a high-risk age group [14]. In addition, having extramarital sexual relationship, being engaged in commercial sex, and suffering from opportunistic infections were significantly associated with HIV seropositivity [15]. Prevalence of latent tuberculosis infection among people who inject drugs was also high and independently associated with HIV infection [16]. An institution-based cross-sectional study showed that having family members treated for pulmonary TB, history of cigarette smoking, WHO HIV clinical stage, and high viral load were associated risk factors of TB among HIV-positive patients [17]. A study in China indicated that many ecological factors, including residents’ income, unemployment rate, and educational level, were closely related to the incidence of tuberculosis [18]. BCG is the only vaccine against TB. It has a positive effect on TB localizations that must be maintained and improved [19].

The lack of updated data on HIV/TB co-infection in China makes assessing the current prevalence and burden of the disease inadequate and results in ineffective policy making. Study period, sample source, place of study, and zone were significantly associated with the co-infection rate [20, 21]. WHO guidelines on collaborative TB/HIV activities and sample size were associated with heterogeneity. Statistically significant subgroup effects with high heterogeneity were observed after subgroup analyses by region, study design, and sample size [22]. In this study, we primarily intend to explore the risk factors, co-infection rate, and subgroup analysis results of HIV/TB co-infection prevalence to provide a theoretical basis for early clinical detection, prevention, and treatment, which is of great significance in the effective reduction of HIV/TB co-infection.

## Materials and methods

### Design and registration

This systematic review and meta-analysis were designed, conducted, and reported based on the Preferred Reporting Items for Systematic Reviews and Meta-Analyses (PRISMA) 2020 guidelines [23]. This study is registered on PROSPERO (Registration number: CRD42022297754).

### Search strategy

Electronic databases, including China National Knowledge Infrastructure (CNKI), Weipu database (VIP), WanFang Data, China Biology Medicine disc (CBM), PubMed^®^, EMBASE, Web of Science, and The Cochrane Library, were searched for HIV/TB co-infection-related publications from inception up to October 2022. Keywords and MeSH terms synonymous with “HIV”, “acquired immunodeficiency syndrome”, “tuberculosis”, “coinfection”, “comorbidity”, “prevalence”, and “risk factors” were used to identify suitable articles in the initial search. References were imported into EndNote™ (Clarivate, Philadelphia, PA, USA) for the initial screening, and duplicates were removed. In addition, reference lists from previous related reviews were screened to ensure a comprehensive search (see Supplementary Materials, Table 1).

### Inclusion and exclusion criteria

The inclusion criteria were as follows: (i) articles that reported Chinese studies with an observational method, which included cross-sectional, case–control, and cohort studies; (ii) study population comprising both adults and children with HIV/AIDS; (iii) studies reporting prevalence and risk factors; (iv) multiple studies from the same sample source included in the study with detailed data; (v) articles written in Chinese or English; (vi) research results related to risk factors expressed as relative risk or odds ratio (OR), provision of a 95% confidence interval (CI). The exclusion criteria were as follows: (i) articles that are periodic reports of a study, with complete results of the study reported in other articles; (ii) special samples that do not represent the general population, such as individuals with drug addiction and prisoners; (iii) studies with incomplete data, unclear data, or obvious errors; (iv) literature reviews, case reports, animal studies, and conference abstracts; (v) studies with a sample size < 100.

### Data extraction

A data extraction table was created using Microsoft Excel (Microsoft Corporation, Redmond, WA, USA). The following data were extracted from the literature: first author, title, publication year, study period, disease diagnostic criteria, study area, study design, number of PLHIV, number of cases of HIV/TB co-infection, sex, age, sample source (hospital/population), and risk factors. Two evaluators (M.L. and Y.Z.) independently screened the literature, extracted data, evaluated the quality and cross-checked the data. Discrepancies were resolved through a centralized discussion or via consultation with a third-party expert (L. X.).

### Quality assessment

The methodological quality of any included cross-sectional studies was assessed according to 11 criteria recommended by the American Agency for Healthcare Research and Quality (see Supplementary Materials, Table 2) [24]. An item was scored ‘1’ if it received ‘yes’ as a response and ‘0’ if it received ‘no’ or ‘unclear’ as a response. Articles were considered low-, medium- and high-quality if the scale scores were 0–3, 4–7, and 8–11, respectively. The methodological quality of any included cohort and case–control studies were assessed using the Newcastle Ottawa scale (see Supplementary Materials, Table 2) [25]. A study with 0–3 stars was considered to have low quality, that with 4–6 stars was considered to have moderate quality, and that with 7–9 stars was considered to have high quality. Articles with a score of 6 or more were included in this meta-analysis. Eighteen low-quality studies were excluded (see Supplementary Materials, List 1).

### Statistical analyses

The combined prevalence and OR of 95% CI were calculated by meta-analysis using STATA^®^ version 17.0 (STATA Corp., College Station, TX, USA). Statistical heterogeneity was assessed using the *I*^2^ test. Sources of heterogeneity were studied by subgroup analysis. This was performed using the sample size, publication time, research area, detection rate, and sample source. Furthermore, to investigate whether the prevalence of HIV/TB co-infection had changed over time, meta-regression analysis was performed to assess whether publication time was a factor for the source of heterogeneity. A funnel plot and Egger’s test were used to analyze the publication bias in the studies included. A *P*-value < 0.05 was considered statistically significant. The trim-and-fill method was used to identify and correct potential publication bias. In addition, sensitivity analyses were conducted to determine whether the inclusion/exclusion of studies led to conceptual differences that affected the final conclusions.

## Results

### Literature screening process and results

**Fig. 1:**
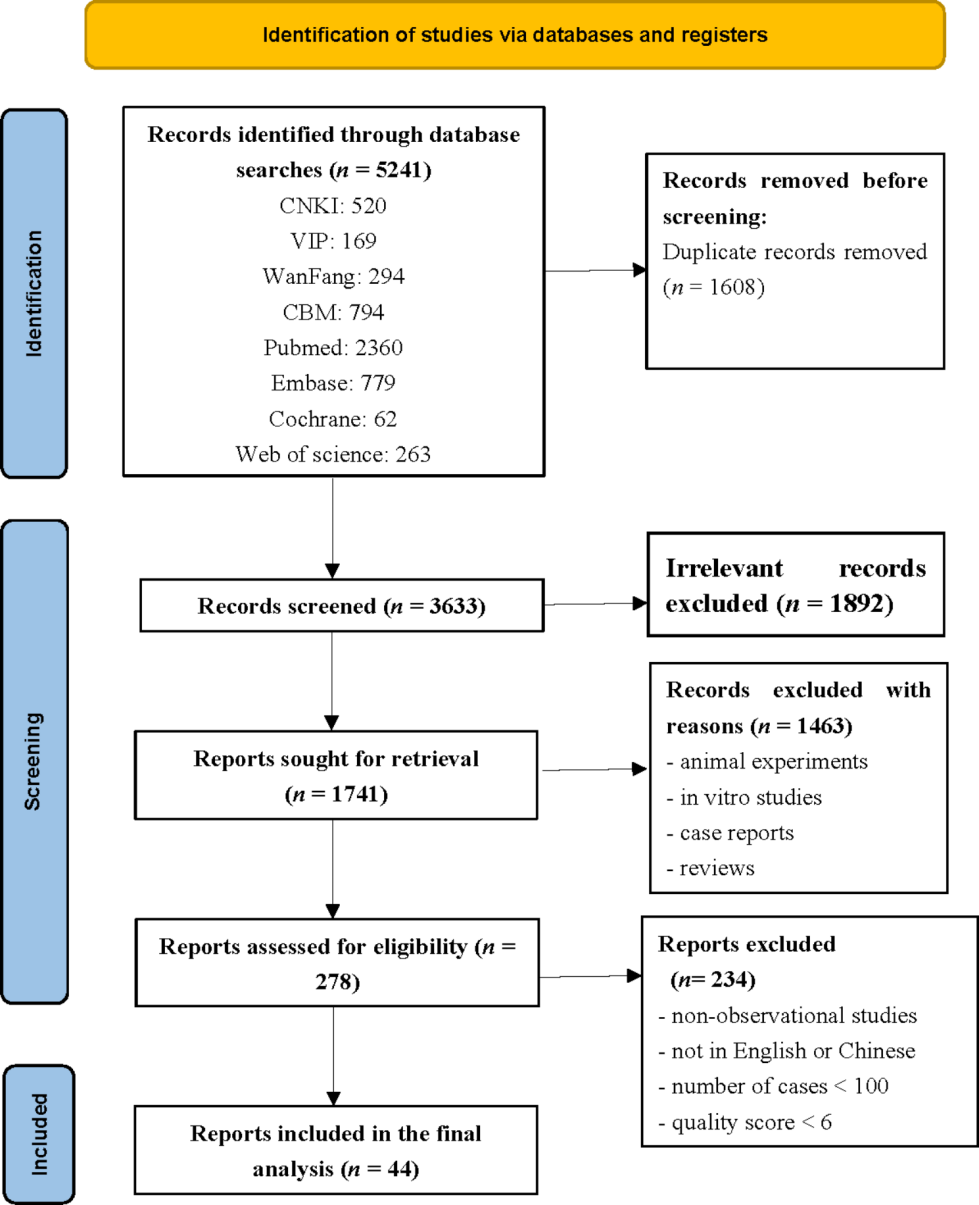
Flow diagram of eligible studies showing the number of citations identified, retrieved, and included in the final meta-analysis undertaken to explore the rate and risk factors of human immunodeficiency virus and tuberculosis co-infection in China

A total of 5241 studies were retrieved by the database searches. After removing duplicates and screening titles, abstracts and full-text, 44 studies (38 Chinese and six English studies) were included (Fig. 1) [26–69].

### Basic characteristics of the included studies

Of these 44 studies, 30 reported the prevalence of TB in PLHIV, involving 94 211 patients [26–29, 31–56]. These 30 studies were published between 2007 and 2022, of which 27 were cross-sectional studies, two were case–control studies, and one was a cohort study [26–29, 31–56]. Only five of the 30 studies were published in English, and the rest were in Chinese. Thirteen studies were reports from hospitals, and seventeen studies reported data acquired from the general population. Studies were selected from China’s four major economic regions [70]. Five studies were from the eastern region [27, 40, 43, 47, 50], ten studies were from the central region [26, 29, 31, 33, 36, 38, 45, 46, 49, 55], 14 studies were from the western region [32, 34, 35, 37, 39, 41, 42, 44, 48, 51–54, 56], and one study was from the mid-western region [28].

The quality scores of all included studies ranged from 6 to 9, indicating that the quality of the included literature in the current meta-analysis was relatively high. The detailed characteristics of the 30 included studies that reported the prevalence of TB in PLHIV are shown in Table 1 [26–29, 31–56]. Nineteen studies reported the risk factors of HIV/TB co-infection, which primarily included CD4^+^ T cell count ≤ 200/µL, smoking, intravenous drug use, unemployment, male sex, and senior citizen status [30, 35, 37, 45, 54, 56–69].

**Table 1:** Baseline characteristics of 30 studies included in a meta-analysis undertaken to explore the rate human immunodeficiency virus (HIV) and tuberculosis (TB) co-infection in China

No.	First author, year	Study period	Study design	Study setting	Region (province/ autonomous region)	Sample size	HIV/TB co-infection	Prevalence	Detection rate
1	Bao, 2010 [26]	2007.10-2008.10	Cross-sectional	Population-based	Hunan	2139	94	4.39%	-
2	Cao, 2021 [55]	2014.1.1-2019.12.31	Cross-sectional	Hospital-based	Anhui	2084	24	1.15%	97.52%
3	Chen, 2009 [27]	2007	Cross-sectional	Population-based	Zhejiang	577	33	5.72%	91.44%
4	Chen, 2017 [54]	2012.10-2013.8	Cross-sectional	Population-based	Guangxi	2987	143	4.80%	99.5%
5	Cheng, 2011 [28]	2006.9-2007.2	Cross-sectional	Hospital-based	Henan, Yunnan, Sichuan and Guangxi	3897	250	6.44%	-
6	Chu, 2010 [29]	2007	Cross-sectional	Population-based	Henan	390	7	1.79%	100%
7	Cui, 2022 [52]	2019–2021	Cohort	Hospital-based	Guangxi	4539	36	0.80%	100%
8	Feng, 2013 [31]	2007.1-2010.12	Cross-sectional	Population-based	Hubei	381	37	9.71%	86.8%
9	He, 2014 [32]	2007.1.31-2012.12.31	Cross-sectional	Hospital-based	Guangxi	2377	96	4.04%	-
10	Huang, 2014 [33]	2007–2011	Cross-sectional	Population-based	Jiangxi	1659	105	6.33%	95%
11	Li, 2010 [34]	2007.1.1-2010.6.30	Cross-sectional	Population-based	Xinjiang	19,453	2104	10.82%	85.34%
12	Li, 2015 [35]	2012–2014	Cross-sectional	Hospital-based	Xinjiang	1195	91	7.60%	83%
13	Liu, 2014 [36]	2007–2011	Cross-sectional	Population-based	Hubei	404	5	1.24%	100%
14	Lv, 2019 [37]	2011.1-2017.12	Case–control	Hospital-based	Sichuan	1366	127	9.30%	100%
15	Mai, 2017 [51]	2006.11-2011.12	Cross-sectional	Hospital-based	Xinjiang	3032	333	11%	100%
16	Mei, 2010 [38]	2007–2008	Cross-sectional	Population-based	Henan	390	50	12.82%	94.36%
17	Nie, 2018 [56]	2004–2015	Case–control	Population-based	Chongqing	19,512	1109	5.68%	95.66%
18	Pan, 2010 [39]	2006–2008	Cross-sectional	Hospital-based	Guizhou	2709	168	6.20%	-
19	Pan, 2022 [53]	2015–2020	Cross-sectional	Population-based	Chongqing	850	46	5.41%	92.79%
20	Qian, 2009 [40]	2007	Cross-sectional	Hospital-based	Shanxi	195	9	4.69%	83%
21	Qu, 2014 [41]	2007.10-2011.12	Cross-sectional	Hospital-based	Guangxi	859	71	8.27%	88.65%
22	Rong, 2014 [42]	2007.1-2011.9	Cross-sectional	Hospital-based	Anhui	1183	27	2.28%	100%
23	Wang, 2010 [43]	2006.9.30-2009.10.1	Cross-sectional	Population-based	Shandong	200	6	3.00%	97.56%
24	Xu, 2009 [44]	2006.10.1-2008.9.30	Cross-sectional	Population-based	Guangxi	10,016	1010	10.08%	64.47%
25	Xu, 2016 [45]	2002.12.16-2012.6.30	Cross-sectional	Population-based	Hunan	205	19	9.27%	97.62%
26	Yin, 2012 [46]	2006.10.1-2010.12.31	Cross-sectional	Hospital-based	Hubei	1409	21	1.49%	92.64%
27	Yin, 2013 [47]	2011.1-2012.6	Cross-sectional	Population-based	Guangdong	208	14	6.70%	14.4%
28	Yuan, 2015 [48]	2010–2013	Cross-sectional	Hospital-based	Guizhou	6071	697	11.48%	86.74%
29	Zhang, 2007 [49]	2006.6-2006.7	Cross-sectional	Population-based	Shanxi	390	36	9.23%	82.63%
30	Zhang, 2015 [50]	2011–2013	Cross-sectional	Population-based	Zhejiang	3534	45	1.27%	95.67%

### Prevalence of HIV/TB co-infection

**Fig. 2:**
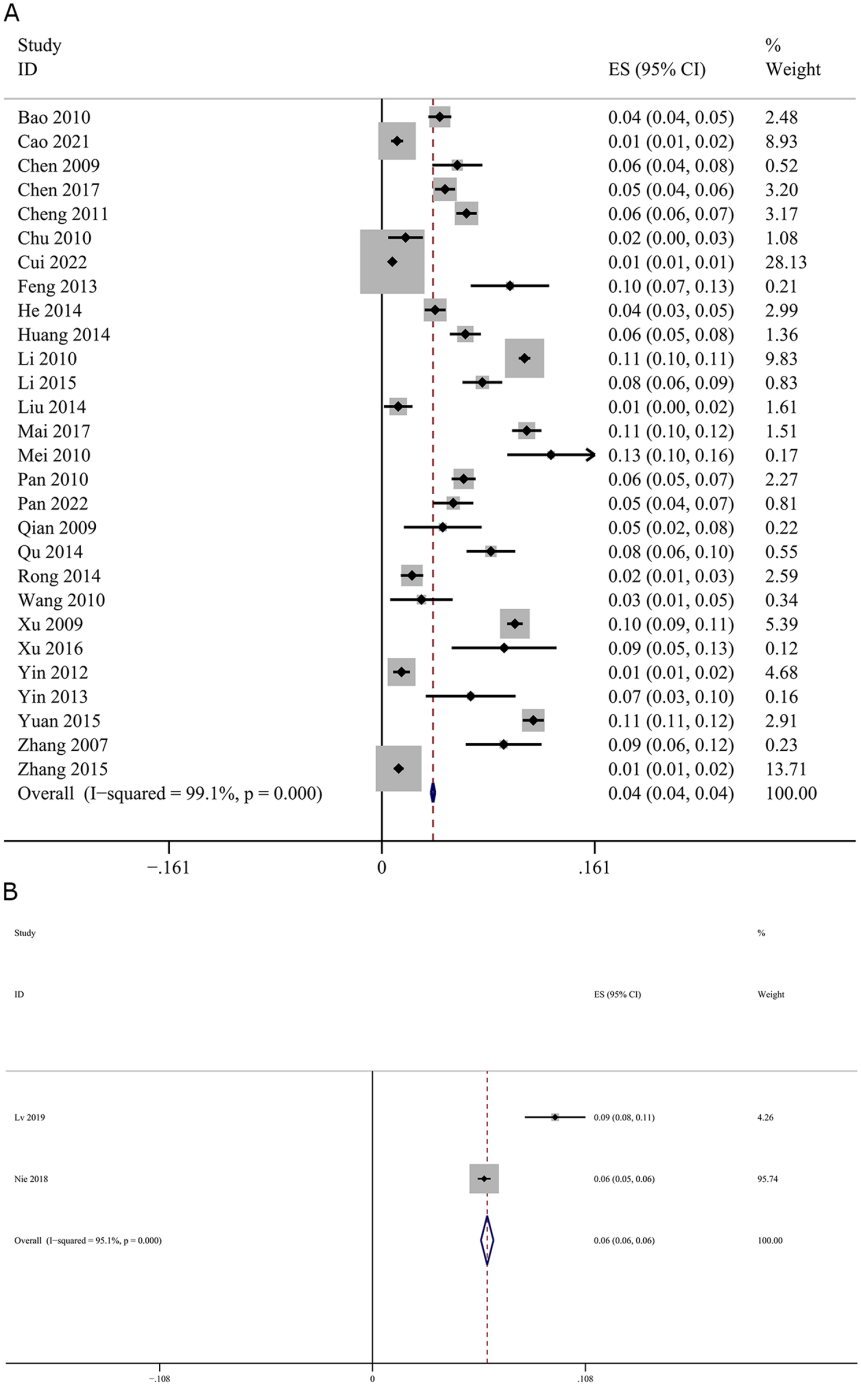
Forest plot showing the pooled prevalence of human immunodeficiency virus and tuberculosis co-infection in China. (**A**) Cross-sectional studies and cohort study; (**B**) Case-control studies. Meta-analyses were constructed using a generic inverse-variance fixed-effects model (for meta-analysis with less than five studies) or random-effects model (for meta-analysis with 5 or more studies)

The heterogeneity of the 27 cross-sectional studies and one cohort study was analyzed (*I*^*2*^ = 99.1% and *P* < 0.01). The random-effects model was used to pool the data. Meta-analysis showed that the prevalence of TB among PLHIV in China was 6.0% (95% CI, 4%, 7%) (Fig. 2A). In case-control studies, the prevalence of TB among PLHIV in China was 6.0% (95% CI, 6%, 6%) (Fig. 2B).

### Subgroup analysis and meta-regression analysis

A subgroup analysis was conducted using the sample size, detection rate, region, publication time, and sample source (Table 2) [26–29, 31–56]. The subgroup analysis showed the following: (i) when the sample size was ≥ 1000, the prevalence of HIV/TB co-infection was 5.8% (95% CI, 4.0%, 7.6%), and when the sample size was < 1000, the prevalence of HIV/TB co-infection was 6.3% (95% CI, 4.3%, 8.3%); (ii) the prevalence of HIV/TB co-infection was 6.6% (95% CI, 4.4%, 8.7%) in 2007–2013 and 5.5% (95% CI, 3.9%, 7.1%) in 2014–2022; (iii) the prevalence of TB among PLHIV in eastern China was 4.1% (95% CI, 1.6%, 6.5%), and the prevalence in central China was 5.1% (95% CI, 3.5%, 6.8%). The prevalence in western China was 7.0% (95% CI, 4.7%, 9.3%), and that in midwest China was 6.4% (95% CI, 5.6%, 7.2%); (iv) the prevalence of TB among PLHIV was 4.4% (95% CI, 1.2%, 7.5%) at a 100% detection rate, 6.7% (95% CI, 4.9%, 8.5%) at a detection rate < 100%, and 5.3% (95% CI, 4.0%, 6.5%) when the detection rate was uncertain; (v) the prevalence of TB among PLHIV was 6.2% (95% CI, 4.4%, 8.1%) when samples were acquired from the population and 5.7% (95% CI, 3.7%, 7.8%) when samples were obtained from hospitals. A meta-regression was also conducted by sorting the manuscript by the publication year of each study. The results showed no significant difference (p = 0.236).

**Table 2:** A subgroup analysis of the prevalence of human immunodeficiency virus and tuberculosis co-infection in China using the sample size, detection rate, region, publication time, and sample source

Variable	No. of studies	Heterogeneity		Prevalence, % (95% CI)
		*I²* (%)	*P*-value	
Sample size				
≥ 1000	18	99.5	*P* < 0.001	5.8 (4.0, 7.6)
< 1000	12	91.2	*P* < 0.001	6.3 (4.3, 8.3)
Publication period				
2007–2013	14	98.4	*P* < 0.001	6.6 (4.4, 8.7)
2014–2022	16	99.0	*P* < 0.001	5.5 (3.9, 7.1)
Geographic region				
Eastern China	5	88.6	*P* < 0.001	4.1 (1.6, 6.5)
Central China	10	95.5	*P* < 0.001	5.1 (3.5, 6.8)
Western China	14	99.5	*P* < 0.001	7.0 (4.7, 9.3)
Midwest China	1	–	–	6.4 (5.6, 7.2)
Detection rate				
100%	6	98.8	*P* < 0.001	4.4 (1.2, 7.5)
< 100%	20	99.1	*P* < 0.001	6.7 (4.9, 8.5)
Uncertain	4	88.4	*P* < 0.001	5.3 (4.0, 6.5)
Study base				
Population	17	98.9	*P* < 0.001	6.2 (4.4, 8.1)
Hospital	13	99.0	*P* < 0.001	5.7 (3.7, 7.8)

### Sensitivity analysis

**Fig. 3:**
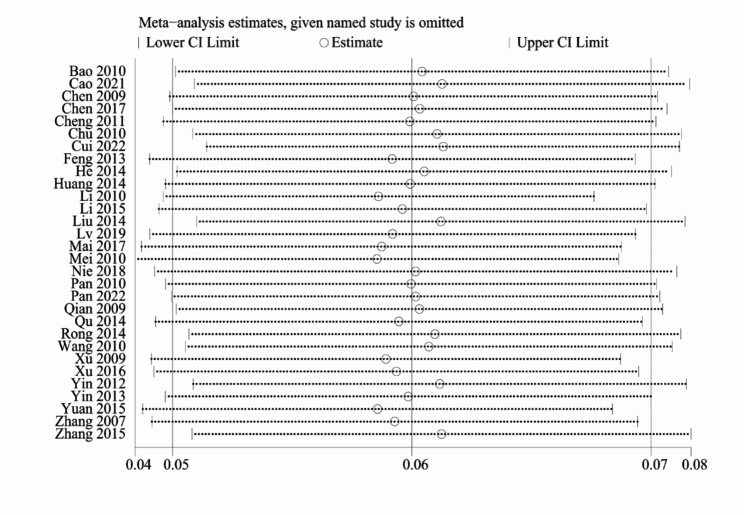
Sensitivity analysis of the pooled prevalence of human immunodeficiency virus and tuberculosis co-infection in China

There was considerable heterogeneity among the results from different studies (*I*^*2*^ = 99.1%, *P* < 0.1). Sensitivity analysis was performed by excluding studies individually. The results did not change significantly (Fig. 3).

### Publication bias analysis

**Fig. 4:**
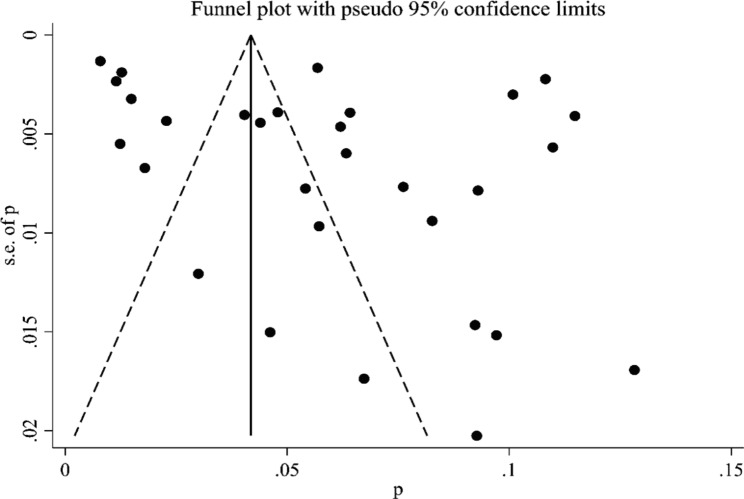
Funnel plot of the pooled prevalence of human immunodeficiency virus and tuberculosis co-infection in China

The Egger’s plot results indicated no publication bias at a *P*-value > 0.05. The funnel plot of the pooled prevalence of HIV/TB co-infection did not show significant asymmetry, and the combination of the results of the Begg’s test (Z = 0.61, *P* = 0.544) and Egger’s test (*t* = 1.84, *P* = 0.076) suggested the absence of publication bias (Fig. 4).

In addition, the results of the subgroup analysis showed a significant publication bias when Egger’s test with a *P*-value of 0.003 was used and the sample size was less than 1000. However, when the sample size was ≥ 1000, publication bias was not detected when Egger’s test with a *P*-value of 0.072 was used.

### Risk factors for HIV/TB co-infection

Fourteen risk factors with more than one occurrence and one protective factor were selected from 19 studies for the meta-analysis (Table 3) [30, 35, 37, 45, 54, 56–69]. The risk factors for TB among PLHIV were as follows (Table 4): CD4^+^ T cell count ≤ 200/µL (OR, 3.062; 95% CI, 1.999, 4.125), smoking (OR, 1.581; 95% CI, 1.299, 1.864), intravenous drug use (OR, 1.862; 95% CI, 1.521, 2.202), unemployment (OR, 1.720; 95% CI, 1.428, 2.013), male sex (OR, 1.623; 95% CI, 1.395, 1.850), senior citizen status (OR, 1.517; 95% CI, 1.319, 1.714), advanced WHO stage (OR, 2.496; 95% CI, 1.539, 3.452), low education level (OR, 1.737; 95% CI, 1.205, 2.268), presence of other opportunistic infections (OR, 2.191; 95% CI, 1.666, 2.716), history of TB (OR, 1.669; 95% CI, 0.674, 2.663), engagement in commercial sex (OR, 2.414; 95% CI, 1.855, 2.972), age 30–45 years (OR, 1.377; 95% CI, 1.037, 1.717), and a long history of HIV (OR, 2.411, 95% CI, 1.766, 3.056). The protective factor was a history of Bacillus Calmette–Guérin (BCG) vaccination (OR, 0.503; 95% CI, 0.246, 0.759). No significant association was observed between low-income families (*P* > 0.05) among PLHIV.

**Table 3:** Baseline characteristics of 19 studies included in the risk factor meta-analysis of human immunodeficiency virus and tuberculosis co-infection in China

No.	First author, year	Study period	Study design	Risk factors	Protective factors	Quality score
1	Chen, 2010 [57]	2007.10.1-2008.10.31	Case–control	2, 4	1	7
2	Chen, 2017 [54]	2012.10-2013.8	Cross-sectional	1, 14	–	7
3	Cui, 2017 [30]	2013–2015	Case–control	1 ,2 ,13	–	7
4	Fan, 2018 [58]	2015.9-2016.8	Case–control	1, 2, 3, 5, 6, 9, 14	–	7
5	Fang, 2020 [59]	2017.1-2019.10	Cohort	4 ,7, 10, 11, 13, 14	1	8
6	He, 2019 [60]	2007.1.31-2012.12.31	Cross-sectional	5, 7, 8, 12	–	8
7	Jiang, 2021 [61]	2014.1-2019.12	Cross-sectional	1, 2, 6, 12	1	7
8	Kou, 2019 [62]	2012.12-2018.3	Case–control	2, 3, 4, 10	1	7
9	Li, 2014 [63]	2008–2012	Cross-sectional	1, 2, 7, 8, 9	–	7
10	Li, 2015 [35]	2012–2014	Cross-sectional	1, 5	–	8
11	Lv, 2019 [37]	2011.1-2017.12	Case–control	1, 3, 9	–	6
12	Meng, 2017 [64]	2013.1-2013.12	Cross-sectional	1, 5, 6	–	7
13	Nie, 2018 [56]	2004–2015	Case–control	1, 5, 7, 11	–	7
14	Wang, 2007 [65]	2005.3-2005.8	Cross-sectional	8	–	7
15	Xu, 2016 [45]	2002.12.16-2012.6.30	Cross-sectional	6	–	8
16	Xu, 2020 [66]	2015.1-2019.11	Cross-sectional	2, 3, 4	1	7
17	Zhang, 2010 [67]	2006.8-2008.3	Cross-sectional	10	–	9
18	Zhou, 2015 [68]	2010.1-2015.1	Case–control	2, 4	1	6
19	Zhang, 2022 [69]	2010.1-2020.12	Case–control	1, 12	1	8

Risk factors: (1) CD4^+^ T cell count ≤ 200/µL; (2) smoking; (3) intravenous drug use; (4) unemployment; (5) male sex; (6) senior citizen status; (7) advanced World Health Organization stage; (8) low family income; (9) low level of education; (10) presence of other opportunistic infections; 11) patients aged 30–45 years; 12) history of tuberculosis; 13) long history of human immunodeficiency virus infection; 14) engagement in commercial sex. Protective factors: 1) History of Bacillus Calmette–Guérin vaccination

**Table 4:** Meta-analysis of associated factors and publication bias examination of 19 studies included in the risk factor meta-analysis of human immunodeficiency virus (HIV) and tuberculosis (TB) co-infection in China

Risk/protective factors	No. of studies	OR (95% CI)	Z	*P*-value	Heterogeneity	
					*P*-value	*I²* (%)
CD4^+^ T cell count ≤ 200/µl	10	3.062 (1.999, 4.125)	5.65	*P* < 0.05	*P* < 0.001	90.3
Smoking	8	1.581 (1.299, 1.864)	10.96	*P* < 0.05	*P* = 0.29	17.7
Intravenous drug use	5	1.862 (1.521, 2.202)	10.72	*P* < 0.05	*P* = 0.101	48.5
Unemployment	5	1.720 (1.428, 2.013)	11.54	*P* < 0.05	*P* = 0.527	0
Male sex	5	1.623 (1.395, 1.850)	13.99	*P* < 0.05	*P* = 0.753	0
Senior citizen status	4	1.517 (1.319, 1.714)	15.06	*P* < 0.05	*P* = 0.879	0
Advanced WHO stage	4	2.496 (1.539, 3.452)	5.12	*P* < 0.05	*P* = 0.014	71.6
Low family income	3	1.827 (–1.310, 4.965)	1.14	*P* = 0.254	*P* = 0.016	82.7
Low level of education	3	1.737 (1.205, 2.268)	6.41	*P* < 0.05	*P* = 0.446	0
Presence of other opportunistic infections	3	2.191 (1.666, 2.716)	8.17	*P* < 0.05	*P* = 0.452	0
History of TB	3	1.669 (0.674, 2.663)	3.29	*P* = 0.001	*P* = 0.019	74.7
Engagement in commercial sex	3	2.414 (1.855, 2.972)	8.47	*P* < 0.05	*P* = 0.625	0
Patients aged 30–45 years	2	1.377 (1.037, 1.717)	7.93	*P* < 0.05	*P* = 0.125	57.6
Long history of HIV	2	2.411 (1.766, 3.056)	7.32	*P* < 0.05	*P* = 0.309	3.2
BCG vaccination history	7	0.503 (0.246, 0.759)	3.84	*P* < 0.05	*P* < 0.001	90.1

OR, odds ratio; CI, confidence interval; WHO, World Health Organization; BCG, Bacillus Calmette–Guérin.

## Discussion

### Analysis of the prevalence of HIV/TB co-infection

Forty-four studies were included in the current meta-analysis, and data from 30 studies were used to analyze the prevalence of HIV/TB co-infection, which was 6.0%. An earlier pooled study published data collected from 1999 to 2010 (OR, 7.2; 95% CI, 4.2, 12.3) [71]. The current findings indicate a downward trend of prevalence compared with the data presented in the aforementioned study. A lower co-infection rate is a positive outcome considering the efforts made by the Chinese government through the Ministry of Health toward reducing HIV- and TB-related morbidity and mortality. These findings are considerably different from the prevalence rates in studies reported in other countries, such as Nepal (9.9%) [72] and Kazakhstan (22%) [73]. This difference may be attributed to race, sexual practices, detection methods, and diagnostic criteria.

The results of statistical analysis are related to the sample size. A sample size less than 50 is considered very poor, a sample size less than 100 is considered poor, a sample size of approximately 200 is considered normal, a sample size of approximately 300 is considered good, a sample size of approximately 500 is considered very good, and a sample size of approximately 1000 is considered ideal; hence, the division by 1000 [74]. Per the results of the current subgroup analysis, a significant publication bias was observed when the sample size was less than 1000. This may be because studies with a small sample size are subject to sampling error and instability. Meta-analyses with small sample sizes can only analyze a subset of the population for which they are conducted, and this subset may deviate from the total population at baseline or in terms of the outcome, i.e., it may lead to prognostic imbalance. When the sample size was ≥ 1000, publication bias was not detected. The frequency of large sample screening should be increased in the future. The prevalence of co-infection in 2014–2022 was significantly lower than that in 2007–2013. UNAIDS reported that 2.1 million [1.9 million–2.4 million] people became newly infected with HIV, and 2.3 million people were added to treatment programs in 2013 [75]. The ONE Campaign, an anti-poverty campaign, said that the human fight against AIDS has reached a milestone in 2013: this marks the first time since antiretroviral medicines were introduced 27 years ago that the treatment of HIV has increased at a greater rate than the incidence of the infection itself [76]. The efficacy of combination therapies (currently referred to as cART instead of HAART) increased from 43% in the mid-90s to 78% in 2010 [77]. Today, HIV infection has become a manageable chronic health condition. This could be one of the reasons for the decline in prevalence.

In recent years, the prevalence rate of HIV/TB co-infection has decreased significantly from 6.6 to 5.5%, which indicates that local governments have paid more attention to this issue. However, the prevalence in the western region was higher than that in the central region, and that in the central region was higher than that in the eastern region. This may be related to the economic conditions of the country. More economically deprived regions have a higher incidence of HIV and a greater risk of concurrent TB [78]. Of the 30 studies included in the current meta-analysis, only six studies had a detection rate of 100%, and those with a detection rate of < 100% had a high pooled prevalence. In the future, awareness and education on the conditions should be improve to achieve comprehensive detection. The prevalence was higher in samples obtained from the population than in those obtained from hospitals. This result could be attributed to the study of selected registered patients with HIV/AIDS.

### Analysis of risk factors of HIV/TB co-infection

Nineteen studies were used to analyze the risk factors of HIV/TB co-infection in the current study. The results showed that CD4^+^ T cell count ≤ 200/µL, smoking, intravenous drug use, unemployment, male sex, senior citizen status, advanced WHO stage, low level of education, and presence of other opportunistic infections increased the risk of TB among PLHIV. CD4^+^ T cells are major immune cells in the human immune system and are the primary target of HIV. A decrease in the CD4^+^ T cell count weakens the immunity; this can lead to various infections. Smoking reduces lung function and destroys the mucous membranes and immune barriers of the respiratory tract. A previous study showed that smoking is associated with disease recurrence and death in patients with TB [79]. Drugs damage the immune system, and individuals with long-term drug addiction have a poor nutritional status and complex interpersonal relationships [80]. Individuals with long-term drug addiction are also more prone to TB infection [81]. Unemployed people and people with low education levels have limited access to health information and lack awareness of HIV transmission routes and hazards. Hence, they are at a greater risk of HIV/TB co-infection, which reflects the positive correlation between poverty and TB from an alternative perspective [82].

The findings of this current meta-analysis demonstrated that patients with advanced WHO stage and a long history of HIV and other opportunistic infections had a higher risk of TB co-infection, which is associated with lower immunity in the later disease stages. People aged 30–45 years are considered young adults; because of work, life circumstances, and other reasons, such patients have considerable population mobility and high sexual activity. Therefore, they are more likely to be infected with *Mycobacterium tuberculosis* [83]. Based on the strong epidemiological association between TB and HIV-1, HIV-1 infection has been hypothesized to increase TB susceptibility by reducing the formation and function of *M. tuberculosis* granulomas [84]. The prevalence of smoking, drinking, and sexual promiscuity is higher in men than in women, which increases the risk of TB in men; this is consistent with evidence from studies conducted in China and abroad [35, 85]. The challenge with the emergence of HIV-associated TB is that attention has been diverted from the fact that the elderly represent the largest pool of individuals with tubercular disease, and the incidence of adverse effects in the elderly is much greater than that in younger patients [86]. Thus, with aging, information about AIDS and TB should be disseminated more widely among the elderly to improve awareness. The current meta-analysis also showed that a history of BCG vaccination is a protective factor for TB among PLHIV. BCG vaccination in neonates can reduce the risk of infection by *M. tuberculosis* and effectively enhance immunity after vaccination to decrease the incidence of TB [87]. BCG vaccination exerts a protective effect, regardless of whether the individual is infected with HIV or not.

### Strengths and limitations of this study

To the best of our knowledge, this current meta-analysis is unique in investigating the prevalence and risk factors of TB among PLHIV/AIDS in China. The findings of this meta-analysis contribute to an understanding of the characteristics of HIV/TB co-infection in various regions of China, which can help formulate corresponding treatment measures. The literature included in this current meta-analysis varied in terms of the study time, study area, sample size, detection rate, and other factors. We performed a meta-regression analysis to assess whether publication time is a source of heterogeneity. The wide economic disparities in different regions of China, which result in a wide variation in the prevalence of HIV/TB co-infection, are also a source of heterogeneity. The included studies had varying samples sizes. Studies with a small sample size may have had sampling error and instability, which may have led to publication bias. Detection rates of less than 100% are also a common problem and do not reflect accurate prevalence rates. All studies included were observational, which may have led to high heterogeneity in the combined literature analysis. Most of the studies selected involved registered cases of HIV/AIDS and the method of TB testing in unregistered patients or patients in the window period remains a problem.

## Conclusions and recommendations

In conclusion, the pooled prevalence of TB among PLHIV was 6.0% in the current meta-analysis. Risk factors for HIV/TB co-infection included CD4^+^ T cell count ≤ 200/µL, smoking, intravenous drug use, unemployment, male sex, senior citizen status, advanced WHO stage, low level of education, and presence of other opportunistic infections. The current meta-analysis showed that the co-infection rate and infection characteristics were distinct in different regions of the country. Therefore, corresponding two-way prevention and control measures should be formulated for different regions in China. For example, for the economically backward Guangxi and Xinjiang areas, dissemination of information on HIV infection and TB should be improved. For transmission via blood transfusion in the Henan area, blood system supervision should be strengthened. In the Yunnan and Hunan regions, which have a high prevalence of drug abuse, AIDS prevention and anti-drug activities should be actively promoted. In the Sichuan and Chongqing areas, which record greater sexual transmission, the understanding of AIDS prevention should be improved, and people should be encouraged to use condoms and reduce risky sexual behavior. Paying more attention to high-risk populations, improving multi-departmental cooperation, using the advantages of multi-disciplinary studies, collectively controlling dual infection, improving the quality of life of patients, and reducing mortality should be the common goals of governments, researchers, and individuals alike.

### Electronic supplementary material

Below is the link to the electronic supplementary material.

Supplementary Material 1

Supplementary Material 2

Supplementary Material 3

## Data Availability

All data generated or analyzed during this study are included in this published article [and its supplementary information files].

## References

[1] World Health Organization. Global HIV Programme. HIV data and statistics, https://www.who.int/teams/global-hiv-hepatitis-and-stis-programmes/hiv/strategic-information/hiv-data-and-statistics (2021, accessed 15 April 2023).

[2] World Health Organization. Tuberculosis & HIV, www.who.int/teams/global-hiv-hepatitis-and-stis-programmes/hiv/treatment/tuberculosis-hiv (2020, accessed 15 Aprily 2023).

[3] Nanta S, Kantipong P, Pathipvanich P (2011). Screening scheme development for active TB prediction among HIV-infected patients. Southeast Asian J Trop Med Public Health.

[4] TB: HIV Study writing Group (2017). One-year mortality of HIV-positive patients treated for rifampicin- and isoniazid-susceptible tuberculosis in Eastern Europe, Western Europe, and Latin America. AIDS.

[5] Hailu T, Yitayal M (2020). Health-Related Quality of Life and Associated factors among adult HIV Mono-Infected and TB/HIV co-infected patients in Public Health Facilities in Northeast Ethiopia: a comparative cross-sectional study. Patient Prefer Adherence.

[6] Guo XY (2022). Anhui AIDS diagnosis and treatment center: left-handed technology, right-handed humanities. President of China Hospital.

[7] World Health Organization. Tuberculosis profile: China, https://worldhealthorg.shinyapps.io/tb_profiles/?_inputs_&entity_type=%22country%22&lan=%22EN%22&iso2=%22CN%22 (2021, accessed 15 April 2023).

[8] Yang Q, Han J, Shen J (2022). Diagnosis and treatment of tuberculosis in adults with HIV. Med (Baltim).

[9] Shen YZ, Li TS (2022). The progress and effectiveness of AIDS prevention and treatment from the changes of China’s AIDS treatment guidelines. Electron J Emerg Infect Dis.

[10] Zhang Y, Jiang T, Li A (2021). Adjunct Therapy for CD4 + T-Cell recovery, inflammation and Immune activation in people living with HIV: a systematic review and Meta-analysis. Front Immunol.

[11] Geremew D, Melku M, Endalamaw A (2020). Tuberculosis and its association with CD4 + T cell count among adult HIV positive patients in ethiopian settings: a systematic review and meta-analysis. BMC Infect Dis.

[12] Adhikari N, Bhattarai RB, Basnet R (2022). Prevalence and associated risk factors for tuberculosis among people living with HIV in Nepal. PLoS ONE.

[13] Wachinou AP, Agodokpessi G, Agbodande A (2018). La tuberculose du sujet âgé en milieu africain: particularités épidémiologiques, diagnostiques et évolutives au Bénin [Tuberculosis in older persons in african setting: Epidemiological, diagnostic and evolutive features]. Rev Pneumol Clin.

[14] Kyaw KWY, Kyaw NTT, Kyi MS (2020). HIV testing uptake and HIV positivity among presumptive tuberculosis patients in Mandalay, Myanmar, 2014–2017. PLoS ONE.

[15] Xu J, Tang W, Cheng S (2014). Prevalence and predictors of HIV among chinese tuberculosis patients by provider-initiated HIV testing and counselling (PITC): a multisite study in South Central of China. PLoS ONE.

[16] Radfar SR, Nematollahi P, Tayeri K (2021). Prevalence of latent tuberculosis infection and HIV among people who inject drugs in Iran. Drug Alcohol Rev.

[17] Toru M, Baye A, Gebeyehu Z (2022). Prevalence, associated factors and rifampicin resistance pattern of pulmonary tuberculosis among HIV-positive patients attending antiretroviral treatment clinic at East Gojjam Zone, Ethiopia: an institution-based cross-sectional study. J Clin Tuberc Other Mycobact Dis.

[18] Zhang Q, Song W, Liu S (2022). An ecological study of tuberculosis incidence in China, from 2002 to 2018. Front Public Health.

[19] Bennasrallah C, Kacem M, Dhouib W (2019). BCG vaccination and tuberculosis prevention: a forty years cohort study, Monastir, Tunisia. PLoS ONE.

[20] Lohiya A, Suliankatchi Abdulkader R, Rath RS (2020). Prevalence and patterns of drug resistant pulmonary tuberculosis in India-A systematic review and meta-analysis. J Glob Antimicrob Resist.

[21] Liu J, Lü B, Yan Y (2013). Meta analysis on the co-infection between Mycobacterium tuberculosis and HIV/AIDS in China. Zhonghua Liu Xing Bing Xue Za Zhi.

[22] Kadia BM, Dimala CA, Fongwen NT (2021). Barriers to and enablers of uptake of antiretroviral therapy in integrated HIV and tuberculosis treatment programmes in sub-saharan Africa: a systematic review and meta-analysis. AIDS Res Ther.

[23] Page MJ, McKenzie JE, Bossuyt PM (2021). The PRISMA 2020 statement: an updated guideline for reporting systematic reviews. BMJ.

[24] Rostom A, Dubé C, Cranney A et al. Celiac Disease. Rockville (MD): Agency for Healthcare Research and Quality (US); 2004 Sep. (Evidence Reports/Technology Assessments, No. 104.). Appendix D. Quality Assessment Forms, https://www.ncbi.nlm.nih.gov/books/NBK35156/ (2004, accessed 20 April 2023).

[25] Yap A, Lopez-Olivo MA, Dubowitz J (2019). Anesthetic technique and cancer outcomes: a meta-analysis of total intravenous versus volatile anesthesia. Can J Anaesth.

[26] Bao CL, Yang HL, Bai LQ (2010). Analysis on TB/HIV two-way screening and the outcome of anti-tuberculosis treatment in 9 counties and districts of Hunan Province. Practical Prev Med.

[27] Chen SH, Wang XM, Liu BD (2009). Analysis of tuberculosis screening among HIV-infected and AIDS patients in Zhejiang Province. Disease Surveillance.

[28] Cheng SM, Zhou L, Liu EY (2011). Cooperation mode and effect of prevention and treatment of Mycobacterium tuberculosis and HIV co-infection. Chin J Antituberculosis.

[29] Chu YZ. Study on opportunistic infection of HIV/AIDS in rural areas of central China. China Medical University; 2010.

[30] Cui Z, Lin M, Nie S (2017). Risk factors associated with tuberculosis (TB) among people living with HIV/AIDS: a pair-matched case-control study in Guangxi, China. PLoS ONE.

[31] Feng WG (2013). Analysis on the effect of Xiangyang Global Fund TB/HIV dual infection prevention and control project. Mod Prev Med.

[32] He J, Lu MF, Lu CJ (2014). Analysis of the effectiveness of the Hengxian Global Fund for tuberculosis and AIDS dual infection prevention and control project. Chin J Prev Med.

[33] Huang WH (2014). Analysis on the results of the fifth round of Global Fund TB/HIV co-infection project in Jiangxi Province. Mod Prev Med.

[34] Li YH, Ma YXT, Wu G. Evaluation of the effect of the fifth round of Global Fund for TB/AIDS co-infection prevention and control projects in Xinjiang: Academic Symposium of the Professional Committee on Tuberculosis Control of the Chinese Antituberculosis Association, Hainan, China, 2010.

[35] Li WG, Zhao L, Zhao H (2015). Epidemiology of HIV-Associated Tuberculosis in Urumqi, China. Transpl Proc.

[36] Liu XY, He LJ, Liu XZ (2014). Analysis of the prevention and treatment of tuberculosis combined with AIDS in Shiyan (2007–2011). Int J Epidemiol Infect Dis.

[37] Lv CR, Tang CX (2019). Epidemiological survey and treatment compliance analysis of AIDS with tuberculosis in Chengdu from 2011 to 2017. Chin Nurs Res.

[38] Mei ZH, Liu Y, Xie JH (2010). Evaluation of the effect of the first phase of the TB/HIV dual infection prevention and treatment project of the Global Fund in Wolong District. China J AIDS STD.

[39] Pan JM, Chen HJ, Li Y (2010). Analysis of the screening situation of TB/AIDS co-infection in Guizhou Province. Chin J Antituberculosis.

[40] Qian HZ, Li Q, Yao H (2009). Tuberculosis co-morbidity and perceptions about health care among HIV-infected plasma donors in rural China. Southeast Asian J Trop Med Public Health.

[41] Qu JX, Li RX, Song XQ et al. Analysis of the prevention and treatment effect of tuberculosis and AIDS dual infection in Binyang County from 2007 to 2011. World Health Digest 2014: 44.

[42] Rong H, Yan JG (2014). Five-year effect evaluation of Jieshou Global Fund TB/HIV dual infection prevention and control project. Anhui J Prev Med.

[43] Wang Y, Li F, Guo XY (2010). Screening analysis of Global Fund TB/HIV dual infection prevention and control project in Shandong Province. Chin J Antituberculosis.

[44] Xu GB, Zhou CM, Cui ZZ (2009). Analysis and evaluation of the fifth round Global Fund TB/HIV double infection project in Guangxi Province. Chin J Antituberculosis.

[45] Xu ZH, Zhang CF, Xiao J (2016). Study on the prevalence and influencing factors of tuberculosis in HIV/AIDS population in Hunan Province. J Chin Physician.

[46] Yin JG, He WH, Lian ZY (2012). Evaluation of the effect of the fifth round of China Global Fund tuberculosis project on Mtb and HIV co-infection in Suizhou City. Chin J Antituberculosis.

[47] Yin JJ, Zhong Q, Zhou L (2013). Evaluation of the effect of Guangdong Global Fund Mtb and HIV co-infection prevention and treatment. Chin J Antituberculosis.

[48] Yuan W, Lei SG, Chen HJ (2015). Analysis of 4-year surveillance results of two-way screening for tuberculosis and AIDS in Guizhou Province. Chin J Health Lab Technolog.

[49] Zhang P, Zhang ZG, Dong XF. Analysis of tuberculosis screening results in HIV/AIDS patients in Yuncheng City. J Practical Med Technol 2007: 5034–5.

[50] Zhang SL, Shan ZL, Mao LQ (2015). Analysis of screening and treatment data for Mycobacterium tuberculosis/HIV co-infection in Wenzhou, Zhejiang Province, 2011–2013. Disease Surveillance.

[51] Maimaiti R, Zhang Y, Pan K (2017). High prevalence and low cure rate of tuberculosis among patients with HIV in Xinjiang, China. BMC Infect Dis.

[52] Cui Z, Huang F, Liang D (2022). Tuberculosis among ambulatory people living with HIV in Guangxi Province, China: a longitudinal study. Int J Environ Res Public Health.

[53] Pan CC, Zhang T, Deng Y (2022). 2015–2020 evaluation of the effectiveness of the prevention and treatment of tuberculosis/AIDS virus dual infection in Wanzhou District, Chongqing. China Prim Health Care.

[54] Chen Z, Li X, Liu Y (2017). Co-infections of tuberculosis, hepatitis B or C viruses in a cohort of people living with HIV/AIDS in China: predictors and sequelae. AIDS Care.

[55] Cao H, Deng XL, Qian B (2021). Analysis on the prevention and treatment of MTB/HIV dual infection in Hefei from 2014 to 2019. Chin J Antitubercul.

[56] Nie H, Li H, Xiao WX (2018). Epidemiological status and related risk factors of AIDS with pulmonary tuberculosis infection in Chongqing area. J Third Military Med Univ.

[57] Chen MS, Yang HL, Chen YF et al. A nested case-control study on the risk factors of tuberculosis in HIV/AIDS population in Hunan Province. Chin J Epidemiol 2010: 151–4. 21215073

[58] Fan GQ, Xu FB, Qi J (2018). Analysis of clinical features and related influencing factors of HIV infection with tuberculosis. Infect Disease Inform.

[59] Fang Z, Huang YS, Wang Q (2020). Epidemiological status and related risk factors of tuberculosis infection among HIV/AIDS patients in Changsha from 2017 to 2019. Occupation and Health.

[60] He B, Nong LP, Li S (2019). Status and influencing factors of HIV/AIDS combined with tuberculosis and tuberculosis infection among HIV/AIDS in rural areas of 5 counties in Nanning City. Guangxi Med J.

[61] Jiang Q, Jiang H, Chen QM (2021). Analysis of risk factors for AIDS with pulmonary tuberculosis in Wanzhou District, Chongqing. Chin J Frontier Health Quarantine.

[62] Kou Y, Ji FB, Fan L (2019). Risk factors for HIV co-infection with tuberculosis. Jiangsu J Prev Med.

[63] Li JH, Jiao YM, Lu Y et al. Analysis of the detection rate and influencing factors of tuberculosis in 199 HIV-infected and AIDS patients in Tianshui City, Gansu Province. Chin J Antituberculosis 2014: 911–3.

[64] Meng SR, Wu FY, Chen XY (2017). Analysis of HIV/TB co-infection in Guangxi and its influencing factors. Chin J New Clin Med.

[65] Wang XW, Liu FY, Dong BQ. Investigation on the detection rate and influencing factors of co-infection in AIDS and tuberculosis patients. Mod Prev Med 2007: 4457–8.

[66] Xu YP, Tian Q, Wang JY (2020). Analysis of risk factors for AIDS complicated with tuberculosis infection. China Med Herald.

[67] Zhang Y, Yu L, Tang ZR (2010). Diagnosis of pulmonary tuberculosis among asymptomatic HIV + patients in Guangxi, China. Chin Med J (Engl).

[68] Zhou JR, Lu XM, Li BL (2015). Analysis of related factors of tuberculosis in HIV/AIDS population. Zhejiang Clin Med J.

[69] Zhang XY, Wang YH, Xiao JP (2022). Analysis on IL-6, hs CRP, TNF-α level and risk factor analysis in AIDS patients with pulmonary tuberculosis. Chin J Trop Med.

[70] Bao H (2010). Analysis of economic differences in China’s four Major Economic Zones: decomposition analysis based on Theil Index. China Dev.

[71] Gao L, Zhou F, Li X (2010). HIV/TB co-infection in mainland China: a meta-analysis. PLoS ONE.

[72] Adhikari N, Bhattarai RB, Basnet R (2022). Prevalence and associated risk factors for tuberculosis among people living with HIV in Nepal. PLoS ONE.

[73] Mukhatayeva A, Mustafa A, Dzissyuk N, Hepatitis B, Hepatitis C (2021). Tuberculosis and sexually-transmitted infections among HIV positive patients in Kazakhstan. Sci Rep.

[74] Yu XN. Meta analysis of Interleukin-10-1082A > G and Interleukin-10-3575T > A gene polymorphisms and susceptibility to lymphoma [D]. Southeast Univ Clin Med, 2014.

[75] UNAIDS. Fast-Track: ending the AIDS epidemic by 2030. https://www.unaids.org/en/resources/presscentre/pressreleaseandstatementarchive/2014/november/20141118_PR_WAD2014report(18 November 2014,accessed 13 July 2023).

[76] Erin Hohlfelder. At the Tipping Point: Tracking Global Resources on AIDS, Volume 3. One Campaign. 2014.https://www.one.org/international/blog/world-aids-day-report-3-steps-to-take-now-weve-reached-the-tipping-point/(2014,accessed 13 July 2023).

[77] Menéndez-Arias L, Delgado R (2022). Update and latest advances in antiretroviral therapy. Trends Pharmacol Sci.

[78] Nattrass N (2009). Poverty, sex and HIV. AIDS Behav.

[79] Chiang CY, Slama K, Enarson DA (2007). Associations between tobacco and tuberculosis. Int J Tuberc Lung Dis.

[80] Nazrul Islam SK, Jahangir Hossain K, Ahmed A (2002). Nutritional status of drug addicts undergoing detoxification: prevalence of malnutrition and influence of illicit drugs and lifestyle. Br J Nutr.

[81] Grenfell P, Baptista Leite R, Garfein R (2013). Tuberculosis, injecting drug use and integrated HIV-TB care: a review of the literature. Drug Alcohol Depend.

[82] Ali ZA, Al-Obaidi MJ, Sameer FO (2022). Epidemiological profile of tuberculosis in Iraq during 2011–2018. Indian J Tuberc.

[83] Moreno V, Espinoza B, Barley K (2017). The role of mobility and health disparities on the transmission dynamics of tuberculosis. Theor Biol Med Model.

[84] Diedrich CR, O’hern J, Wilkinson RJ (2016). HIV-1 and the Mycobacterium tuberculosis granuloma: a systematic review and meta-analysis. Tuberculosis (Edinb).

[85] Tran NB, Houben RM, Hoang TQ (2007). HIV and tuberculosis in Ho Chi Minh City, Vietnam, 1997–2002. Emerg Infect Dis.

[86] Davies PD (1996). Tuberculosis in the elderly. Epidemiology and optimal management. Drugs Aging.

[87] Lv GB, Li H (2011). Discussion on tuberculosis prevention, diagnosis and treatment standard. Seek Med Ask Med (Second half Month).

