# Supplementary material for: Prevalence and risk factors of tuberculosis among people living with HIV/AIDS in China: a systematic review and meta-analysis
Source: BMC Infect Dis. 2023 Sep 6;23:584. doi: 10.1186/s12879-023-08575-4 (PMC10481577; doi:10.1186/s12879-023-08575-4)
Supplement: Supplementary file 1 — Supplementary Material 1 [file 12879_2023_8575_MOESM1_ESM.pdf]

Supplementary Table 1

Search Strategy for Electronic Databases

Database	Search Number	Search Strategy	Results
PubMed	#1	"Acquired Immunodeficiency Syndrome"[Mesh]	77,952
	#2	"Immunologic Deficiency Syndrome, Acquired" [Title/Abstract] OR "Acquired Immune Deficiency Syndrome"[Title/Abstract] OR "Acquired Immuno-Deficiency Syndrome*"[Title/Abstract] OR "Acquired Immuno Deficiency Syndrome"[Title/Abstract] OR "Immuno-Deficiency Syndrome*, Acquired" [Title/Abstract] OR "Syndrome*, Acquired Immuno-Deficiency"[Title/Abstract] OR "Immunodeficiency Syndrome*, Acquired"[Title/Abstract] OR "Acquired Immunodeficiency Syndromes"[Title/Abstract] OR "Syndrome*, Acquired Immunodeficiency" [Title/Abstract] OR "AIDS" [Title/Abstract]	171,068
	#3	"HIV"[Mesh]	106,005
	#4	((((((((((((((((((((((Human Immunodeficiency Virus* [Title/Abstract]) OR (Immunodeficiency Virus*, Human[Title/Abstract])) OR (Virus*, Human Immunodeficiency[Title/Abstract])) OR (Human T Cell Lymphotropic Virus Type III[Title/Abstract])) OR (Human T-Cell Lymphotropic Virus Type III[Title/Abstract])) OR (Human T-Cell Leukemia Virus Type III[Title/Abstract])) OR (Human T Cell Leukemia Virus Type III[Title/Abstract])) OR (LAV-HTLV-III[Title/Abstract])) OR (Lymphadenopathy-Associated Virus* [Title/Abstract])) OR (Lymphadenopathy Associated Virus[Title/Abstract])) OR (Virus*, Lymphadenopathy-Associated[Title/Abstract])) OR (Human T Lymphotropic Virus Type III[Title/Abstract])) OR (Human T-Lymphotropic Virus Type III[Title/Abstract])) OR (AIDS Virus*[Title/Abstract])) OR (Virus*, AIDS[Title/Abstract])) OR (Acquired Immune Deficiency Syndrome Virus[Title/Abstract])) OR (Acquired Immunodeficiency Syndrome Virus[Title/Abstract])) OR (HTLV-III[Title/Abstract])	98,339
	#5	#1 OR #2 OR #3 OR #4	318,876
	#6	"Tuberculosis"[Mesh]	203,495
	#7	"Tuberculoses" [Title/Abstract] OR "Kochs Disease" [Title/Abstract] OR "Koch's Disease" [Title/Abstract] OR "Koch Disease" [Title/Abstract] OR "Mycobacterium tuberculosis Infection*" [Title/Abstract] OR "Infection*, Mycobacterium tuberculosis" [Title/Abstract]	3,367

	#8	"Tuberculoses" [Title/Abstract] OR "Kochs Disease" [Title/Abstract] OR "Koch's Disease" [Title/Abstract] OR "Koch Disease" [Title/Abstract] OR "Mycobacterium tuberculosis Infection*" [Title/Abstract] OR "Infection*, Mycobacterium tuberculosis" [Title/Abstract] OR ("Tuberculosis"[Mesh])	204,232
	#9	#5 AND #8	9,317
	#10	"Prevalence"[Mesh]	335,562
	#11	((((((Prevalences[Title/Abstract]) OR (Period Prevalence[Title/Abstract])) OR (Period Prevalences[Title/Abstract])) OR (Prevalence, Period[Title/Abstract])) OR (Point Prevalence[Title/Abstract])) OR (Point Prevalences[Title/Abstract])) OR (Prevalence, Point[Title/Abstract])	23,213
	#12	(relative[Title/Abstract] AND risk*[Title/Abstract]) OR (relative risk[Text Word]) OR risks[Text Word] OR cohort studies[MeSH:noexp] OR (cohort[Title/Abstract] AND stud*[Title/Abstract]) OR cross-sectional studies[MeSH:noexp] OR (cross-sectional[Title/Abstract] AND stud*[Title/Abstract]) OR case-control studies[MeSH:noexp] OR (case-control[Title/Abstract] AND stud*[Title/Abstract])	1,952,829
	#13	#10 OR #11 OR #12	2,180,020
	#14	#9 AND #13	2,360
Embase	#1	'human immunodeficiency virus'/exp	214,231
	#2	'immunodeficiency virus*, human':ti,ab OR 'virus*, human immunodeficiency':ti,ab OR 'human t cell lymphotropic virus type iii':ti,ab OR 'human t-cell lymphotropic virus type iii':ti,ab OR 'human t-cell leukemia virus type iii':ti,ab OR 'human t cell leukemia virus type iii':ti,ab OR 'lav-htlv-iii':ti,ab OR 'lymphadenopathy-associated virus*':ti,ab OR 'lymphadenopathy associated virus':ti,ab OR 'virus*, lymphadenopathy-associated':ti,ab OR 'human t lymphotropic virus type iii':ti,ab OR 'human t-lymphotropic virus type iii':ti,ab OR 'aids virus*':ti,ab OR 'virus*, aids':ti,ab OR 'acquired immune deficiency syndrome virus':ti,ab OR 'acquired immunodeficiency syndrome virus':ti,ab OR 'htlv-iii':ti,ab	3,885
	#3	'acquired immune deficiency syndrome'/exp	152,688
	#4	'immunologic deficiency syndrome, acquired':ti,ab OR 'acquired immune deficiency syndrome':ti,ab OR 'acquired immuno-deficiency syndrome*':ti,ab OR 'acquired immuno deficiency syndrome':ti,ab OR 'immuno-deficiency syndrome*,acquired':ti,ab OR 'syndrome*,acquired immuno-deficiency':ti,ab OR 'immunodeficiency syndrome*, acquired':ti,ab OR 'acquired immunodeficiency syndromes':ti,ab OR 'syndrome*, acquired immunodeficiency':ti,ab OR 'aids':ti,ab	190,496

	#5	#1 OR #2 OR #3 OR #4	420,604
	#6	'tuberculosis'/exp	289,990
	#7	'tuberculoses':ti,ab OR 'kochs disease':ti,ab OR 'koch disease':ti,ab OR 'mycobacterium tuberculosis infection*':ti,ab OR 'infection*, mycobacterium tuberculosis':ti,ab	3,959
	#8	#6 OR #7	290,480
	#9	'prevalence'/exp	917,465
	#10	'period prevalence*':ti,ab OR 'prevalence, period':ti,ab OR 'point prevalence*':ti,ab OR 'prevalence, point':ti,ab	10,362
	#11	'risk factor'/exp	1,256,250
	#12	'factor, risk':ti,ab OR 'risk factor':ti,ab OR 'social risk factors*':ti,ab OR 'factor*, social risk':ti,ab OR 'risk factor*, social':ti,ab OR 'health correlates':ti,ab OR 'correlates, health':ti,ab OR 'population* at risk':ti,ab OR 'risk scores*':ti,ab OR 'score, risk':ti,ab OR 'risk factor scores*':ti,ab OR 'score, risk factor':ti,ab	398,006
	#13	#9 OR #10 OR #11 OR #12	2,153,871
	#14	#5 AND #8 AND #13	4,202
	#15	#14 AND ('case control study'/de OR 'cohort analysis'/de OR 'cross sectional study'/de OR 'observational study'/de)	779
Cochrane	#1	MeSH descriptor: [Acquired Immunodeficiency Syndrome] explode all trees	2,054
	#2	(Immunologic Deficiency Syndrome, Acquired):ti,ab,kw OR (Acquired Immune Deficiency Syndrome):ti,ab,kw OR (Acquired Immuno Deficiency Syndrome):ti,ab,kw OR (Immuno-Deficiency Syndrome*, Acquired):ti,ab,kw OR (Syndrome*, Acquired Immuno-Deficiency):ti,ab,kw OR (Immunodeficiency Syndrome*, Acquired):ti,ab,kw OR (Acquired Immunodeficiency Syndromes):ti,ab,kw OR (Syndrome*, Acquired Immunodeficiency):ti,ab,kw OR (AIDS):ti,ab,kw	11,825
	#3	MeSH descriptor: [HIV] explode all trees	3,275
	#4	(immunodeficiency virus*, human):ti,ab,kw OR (virus*, human immunodeficiency):ti,ab,kw OR (human t cell lymphotropic virus type iii):ti,ab,kw OR (human t-cell lymphotropic virus type iii):ti,ab,kw OR (human t-cell leukemia virus type iii):ti,ab,kw OR (human t cell leukemia virus type iii):ti,ab,kw OR (lav-htlv-iii):ti,ab,kw OR (lymphadenopathy-associated virus*):ti,ab,kw OR (lymphadenopathy associated virus):ti,ab,kw OR (virus*, lymphadenopathy-associated):ti,ab,kw OR (human t lymphotropic virus type iii):ti,ab,kw OR (human t-lymphotropic virus type iii):ti,ab,kw OR (aids virus*):ti,ab,kw OR (virus*, aids):ti,ab,kw OR (acquired immune deficiency syndrome virus):ti,ab,kw OR (acquired immunodeficiency syndrome virus):ti,ab,kw OR (htlv-iii):ti,ab,kw	3,285
	5	#1 or #2 or #3 or #4	14,768

	#6	MeSH descriptor: [Tuberculosis] explode all trees	2,637
	#7	(Tuberculoses):ti,ab,kw OR (Kochs Disease):ti,ab,kw OR (Koch's Disease):ti,ab,kw OR (Koch Disease):ti,ab,kw OR (Mycobacterium tuberculosis Infection*):ti,ab,kw OR (Infection*, Mycobacterium tuberculosis):ti,ab,kw	596
	#8	#6 or #7	2,946
	#9	MeSH descriptor: [Prevalence] explode all trees	4,978
	#10	(period prevalence*):ti,ab,kw OR (prevalence, period):ti,ab,kw OR (point prevalence*):ti,ab,kw OR (prevalence, point):ti,ab,kw	9,571
	#11	MeSH descriptor: [Risk Factors] explode all trees	26,359
	#12	(Factor, Risk):ti,ab,kw OR (Risk Factor):ti,ab,kw OR (Social Risk Factors*):ti,ab,kw OR (Factor*, Social Risk):ti,ab,kw OR (Risk Factor*, Social):ti,ab,kw OR (Health Correlates):ti,ab,kw OR (Correlates, Health):ti,ab,kw OR (population* at Risk):ti,ab,kw OR (Risk Scores*):ti,ab,kw OR (Score, Risk):ti,ab,kw OR (Risk Factor Scores*):ti,ab,kw OR (Score, Risk Factor):ti,ab,kw	119,682
	#13	#9 or #10 or #11 or #12	143,582
	#14	#5 and #8 and #13	62
Web of science	#1	TS=((Acquired Immunodeficiency Syndrome) OR (Immunologic Deficiency Syndrome, Acquired) OR (Acquired Immune Deficiency Syndrome) OR (Acquired Immuno Deficiency Syndrome) OR (Immuno-Deficiency Syndrome*, Acquired) OR (Syndrome*, Acquired Immuno-Deficiency) OR (Immunodeficiency Syndrome*, Acquired) OR (Acquired Immunodeficiency Syndromes) OR (Syndrome*, Acquired Immunodeficiency) OR (AIDS) OR (HIV) OR (Human Immunodeficiency Virus*) OR (Immunodeficiency Virus*, Human) OR (Virus*, Human Immunodeficiency) OR (Human T Cell Lymphotropic Virus Type III) OR (Human T-Cell Lymphotropic Virus Type III) OR (Human T-Cell Leukemia Virus Type III) OR (Human T Cell Leukemia Virus Type III) OR (LAV-HTLV-III) OR (Lymphadenopathy-Associated Virus*) OR (Lymphadenopathy Associated Virus) OR (Virus*, Lymphadenopathy-Associated) OR (Human T Lymphotropic Virus Type III) OR (Human T-Lymphotropic Virus Type III) OR (AIDS Virus*) OR (Virus*, AIDS) OR (Acquired Immune Deficiency Syndrome Virus) OR (Acquired Immunodeficiency Syndrome Virus) OR (HTLV-III))	737,536
	#2	TS=((Tuberculoses) OR (Kochs Disease) OR (Koch's Disease) OR (Koch Disease) OR (Mycobacterium tuberculosis Infection*) OR (Infection*, Mycobacterium tuberculosis) OR (Tuberculosis))	129,454
	#3	TS=((prevalence*) OR (Period Prevalence*) OR (Prevalence, Period) OR (Point Prevalence*) OR (Prevalence, Point) OR (Factor, Risk) OR (Risk Factor) OR (Social Risk Factors*) OR	2,328,392

	(Factor*, Social Risk) OR (Risk Factor*, Social) OR (Health Correlates) OR (Correlates, Health) OR (population* at Risk) OR (Risk Scores*) OR (Score, Risk) OR (Risk Factor Scores*) OR (Score, Risk Factor) OR (Risk Factors))	
#4	#3 AND #2 AND #1	6,745
#5	#4 and PEOPLES R CHINA	263
