# Supplementary material for: Prevalence and risk factors of tuberculosis among people living with HIV/AIDS in China: a systematic review and meta-analysis
Source: BMC Infect Dis. 2023 Sep 6;23:584. doi: 10.1186/s12879-023-08575-4 (PMC10481577; doi:10.1186/s12879-023-08575-4)
Supplement: Supplementary file 2 — Supplementary Material 2 [file 12879_2023_8575_MOESM2_ESM.doc]

| **Item** | **Yes** | **No** | **Unclear** |
| --- | --- | --- | --- |
| 1) Define the source of information (survey, record review) |  |  |  |
| 2) List inclusion and exclusion criteria for exposed and unexposed subjects (cases and controls) or refer to previous publications |  |  |  |
| 3) Indicate time period used for identifying patients |  |  |  |
| 4) Indicate whether or not subjects were consecutive if not population-based |  |  |  |
| 5) Indicate if evaluators of subjective components of study were masked to other aspects of the status of the participants |  |  |  |
| 6) Describe any assessments undertaken for quality assurance purposes (e.g., test/retest of primary outcome measurements) |  |  |  |
| 7) Explain any patient exclusions from analysis |  |  |  |
| 8) Describe how confounding was assessed and/or controlled. |  |  |  |
| 9) If applicable, explain how missing data were handled in the analysis |  |  |  |
| 10) Summarize patient response rates and completeness of data collection |  |  |  |
| 11) Clarify what follow-up, if any, was expected and the percentage of patients for which incomplete data or follow-up was obtained |  |  |  |

**AHRQ cross-sectional study quality evaluation scale**

**CODING MANUAL FOR CASE-CONTROL STUDIES**

***SELECTION***

**1) Is the Case Definition Adequate?**

a) Requires some independent validation (e.g. >1 person/record/time/process to extract information, or reference to primary record source such as x-rays or medical/hospital records) ☆

b) Record linkage (e.g. ICD codes in database) or self-report with no reference to primary record

c) No description

**2) Representativeness of the Cases**

a) All eligible cases with outcome of interest over a defined period of time, all cases in a defined catchment area, all cases in a defined hospital or clinic, group of hospitals, health maintenance organisation, or an appropriate sample of those cases (e.g. random sample) ☆

b) Not satisfying requirements in part (a), or not stated.

**3) Selection of Controls This item assesses whether the control series used in the study is derived from the same population as the cases and essentially would have been cases had the outcome been present.**

a) Community controls (i.e. same community as cases and would be cases if had outcome) ☆

b) Hospital controls, within same community as cases (i.e. not another city) but derived from a hospitalised population

c) No description

**4) Definition of Controls**

a) If cases are first occurrence of outcome, then it must explicitly state that controls have no history of this outcome. If cases have new (not necessarily first) occurrence of outcome, then controls with previous occurrences of outcome of interest should not be excluded. ☆

b) No mention of history of outcome

***COMPARABILITY***

1. **Comparability of Cases and Controls on the Basis of the Design or Analysis**

A maximum of 2 stars can be allotted in this category

Either cases and controls must be matched in the design and/or confounders must be adjusted for in the analysis. Statements of no differences between groups or that differences were not statistically significant are not sufficient for establishing comparability.

Note: If the odds ratio for the exposure of interest is adjusted for the confounders listed, then the groups will be considered to be comparable on each variable used in the adjustment.

There may be multiple ratings for this item for different categories of exposure (e.g. ever vs. never, current vs. previous or never)

Age =☆, Other controlled factors = ☆

***EXPOSURE***

1. **Ascertainment of Exposure**

Allocation of stars as per rating sheet

1. **Non-Response Rate**

Allocation of stars as per rating sheet

**CODING MANUAL FOR COHORT STUDIES**

***SELECTION***

1. **Representativeness of the Exposed Cohort**

Item is assessing the representativeness of exposed individuals in the community, not the representativeness of the sample of women from some general population. For example, subjects derived from groups likely to contain middle class, better educated, health oriented women are likely to be representative of postmenopausal estrogen users while they are not representative of all women (e.g. members of a health maintenance organisation (HMO) will be a representative sample of estrogen users. While the HMO may have an under-representation of ethnic groups, the poor, and poorly educated, these excluded groups are not the predominant users users of estrogen).

Allocation of stars as per rating sheet

1. **Selection of the Non-Exposed Cohort**

Allocation of stars as per rating sheet

1. **Ascertainment of Exposure**

Allocation of stars as per rating sheet

1. **Demonstration That Outcome of Interest Was Not Present at Start of Study**

In the case of mortality studies, outcome of interest is still the presence of a disease/ incident, rather than death. That is to say that a statement of no history of disease or incident earns a star.

***COMPARABILITY***

1. **Comparability of Cohorts on the Basis of the Design or Analysis**

A maximum of 2 stars can be allotted in this category Either exposed and non-exposed individuals must be matched in the design and/or confounders must be adjusted for in the analysis. Statements of no differences between groups or that differences were not statistically significant are not sufficient for establishing comparability. Note: If the relative risk for the exposure of interest is adjusted for the confounders listed, then the groups will be considered to be comparable on each variable used in the adjustment.

There may be multiple ratings for this item for different categories of exposure (e.g. ever vs. never, current vs. previous or never)

Age = ☆, Other controlled factors = ☆

***OUTCOME***

**1) Assessment of Outcome**

For some outcomes (e.g. fractured hip), reference to the medical record is sufficient to satisfy the requirement for confirmation of the fracture. This would not be adequate for vertebral fracture outcomes where reference to x-rays would be required.

a) Independent or blind assessment stated in the paper, or confirmation of the outcome by reference to secure records (x-rays, medical records, etc.) ☆

b) Record linkage (e.g. identified through ICD codes on database records) ☆

c) Self-report (i.e. no reference to original medical records or x-rays to confirm the outcome) d) No description.

**2) Was Follow-Up Long Enough for Outcomes to Occur**

An acceptable length of time should be decided before quality assessment begins (e.g. 5 yrs. for exposure to breast implants)

**3) Adequacy of Follow Up of Cohorts**

This item assesses the follow-up of the exposed and non-exposed cohorts to ensure that losses are not related to either the exposure or the outcome.

Allocation of stars as per rating sheet
