# Supplementary material for: Prevalence and risk factors of tuberculosis among people living with HIV/AIDS in China: a systematic review and meta-analysis
Source: BMC Infect Dis. 2023 Sep 6;23:584. doi: 10.1186/s12879-023-08575-4 (PMC10481577; doi:10.1186/s12879-023-08575-4)
Supplement: Supplementary file 3 — Supplementary Material 3 [file 12879_2023_8575_MOESM3_ESM.docx]

**List 1. List of poor quality articles excluded from this study**

[1] Dai Y, Li TS, Wang AX, et al. Analysis of the clinical characteristics of 143 Chinese AIDS patients detected at first diagnosis[J]. Journal of the Chinese Academy of Medical Sciences,2006(05):651-654.

[2] Li SF, Zhao DY, Liu GZ, et al. Investigation and analysis of HIV-associated tuberculosis infection--with clinical observation of 18 cases[J]. Henan Journal of Preventive Medicine,2008(03):175-176.

[3] Ling XL, Hu HZ. Analysis of clinical characteristics associated with patients with HIV infection secondary to AIDS in general hospitals[J]. Marker immunoassay and clinical,2020,27(01):82-85+89.

[4] Luo XL, Huang WH, Wei SQ, et al. Pathogen distribution and CD4 levels in elderly patients with acquired immunodeficiency syndrome co-infection in Guangxi[J]. Chinese Journal of Gerontology,2014,34(08):2102-2103.

[5] Ma LX, Yang H, Zhang QJ, et al. Study on surveillance and prevention of TB/HIV dual infection in Shijiazhuang City[J]. Medical Animal Prevention,2013,29(01):104-105.

[6] Meng WY, Li XK, Li XC, et al. Clinical and therapeutic analysis of 105 cases of HIV infection/AIDS [J]. Chinese Journal of Dermatologic Venereology,2009,23(10):643-644.

[7] Pi XB, Wang JH, Ouyang JX. Analysis of HIV antibody screening results in 441,867 inpatients in a hospital in Foshan from 2006 to 2012 [J]. Chinese Journal of Dermatologic Venereology,2014,28(04):379-381.

[8] Wang M, Fan XG, Xu D, et al. Clinical characteristics and spectrum of opportunistic infections in 388 patients with HIV/AIDS[J]. Practical preventive medicine,2010,17(01):168-171.

[9] Wei FB, Lu RZ. Screening analysis of Mycobacterium tuberculosis in HIV/AIDS combined with tuberculosis[J]. Journal of Guangxi Medical University,2009,26(04):573-574.

[10] Xu JG, Fang J, Huang YL, et al. Network reporting of other infectious diseases in patients with HIV/AIDS[J]. Modern medicine and health,2019,35(21):3285-3287+3290.

[11] Yi F, Li Y, Cai MQ, et al. Statistical analysis of HIV-infected patients with other co-morbidities in southern Yunnan[J]. Southwest Defense Medicine,2011,21(04):395-397.

[12] Zhang HF, Deng GB, Huo Q, et al. Clinical characteristics of 226 cases of elderly patients hospitalized with acquired immunodeficiency syndrome[J]. Chinese Journal of Infection and Chemotherapy,2021,21(03):288-291.

[13] Zhang L, Chen YP, Li Q. HBV, HCV, TP and TB test results in HIV-infected patients [J]. Primary Medical Forum,2021,25(26):3792-3794.

[14] Xu CY, Zhang YF, Zhu YJ, et al. Study on the correlation between lymphocyte counts and opportunistic infections in HIV/AIDS patients in Guangxi[J]. Journal of Taishan Medical College,2013,34(11):858-860.

[15] Yao Y X, Huang C Y, Zhong M, et al. Analysis of clinical characteristics of AIDS and tuberculosis in a center[J]. Chongqing Medicine,2014,43(35):4806-4807.

[16] Wang YF, Wang Y, Xie LZ, et al. Analysis of 183 cases of tuberculosis screening in HIV-positive patients[J]. Henan Journal of Preventive Medicine,2007(01):29-30.

[17] Xu YY, Huo S, Wu XH, et al. Analysis of clinical characteristics of 132 cases of opportunistic HIV infection [J]. Health Soft Science,2007(06):513-516.

[18] Zhang GL, Jiao M. Analysis of the characteristics and influencing factors of HIV infection combined with tuberculosis[J]. Health,2020(20):164,166.
